# Linear and Volumetric Polyethylene Wear Patterns after Primary Cruciate-Retaining Total Knee Arthroplasty Failure: An Analysis Using Optical Scanning and Computer-Aided Design Models

**DOI:** 10.3390/ma17205007

**Published:** 2024-10-13

**Authors:** Matej Valič, Ingrid Milošev, Vesna Levašič, Mateja Blas, Eva Podovšovnik, Jaka Koren, Rihard Trebše

**Affiliations:** 1 Valdoltra Orthopaedic Hospital, Jadranska cesta 31, 6280 Ankaran, Slovenia; ingrid.milosev@ob-valdoltra.si (I.M.); vesna.levasic@ob-valdoltra.si (V.L.); mateja.blas@ob-valdoltra.si (M.B.); eva.podovsovnik@ob-valdoltra.si (E.P.); rihard.trebse@ob-valdoltra.si (R.T.); 2 Faculty of Medicine, University of Ljubljana, Vrazov trg 2, 1000 Ljubljana, Slovenia; 3 Jožef Stefan Institute, Jamova cesta 39, 1000 Ljubljana, Slovenia; 4 Faculty of Tourism Studies-Turistica, University of Primorska, Obala 11a, 6320 Portorož, Slovenia; 5 Faculty of Electrical Engineering, University of Ljubljana, Tržaška cesta 25, 1000 Ljubljana, Slovenia; jkkoren55@gmail.com

**Keywords:** total knee reconstruction, implant retrieval analysis, failure analysis, polyethylene wear, volumetric wear, articular surface wear distribution, endoprosthesis, orthopedic surgery

## Abstract

(1) Background: Analyses of retrieved inserts allow for a better understanding of TKA failure mechanisms and the detection of factors that cause increased wear. The purpose of this implant retrieval study was to identify whether insert volumetric wear significantly differs among groups of common causes of total knee arthroplasty failure, whether there is a characteristic wear distribution pattern for a common cause of failure, and whether nominal insert size and component size ratio (femur-to-insert) influence linear and volumetric wear rates. (2) Methods: We digitally reconstructed 59 retrieved single-model cruciate-retaining inserts and computed their articular load-bearing surface wear utilizing an optical scanner and computer-aided design models as references. After comprehensively reviewing all cases, each was categorized into one or more of the following groups: prosthetic joint infection, osteolysis, clinical loosening of the component, joint malalignment or component malposition, instability, and other isolated causes. The associations between volumetric wear and causes of failure were estimated using a multiple linear regression model adjusted for time in situ. Insert linear penetration wear maps from the respective groups of failure were further processed and merged to create a single average binary image, highlighting a potential wear distribution pattern. The differences in wear rates according to nominal insert size (small vs. medium vs. large) and component size ratio (≤1 vs. >1) were tested using the Kruskal–Wallis test and the Mann–Whitney test, respectively. (3) Results: Patients with identified osteolysis alone and those also with clinical loosening of the component had significantly higher volumetric wear when compared to those without both causes (*p* = 0.016 and *p* = 0.009, respectively). All other causes were not significantly associated with volumetric wear. The instability group differentiated from the others with a combined peripheral antero-posterior wear distribution. Linear and volumetric wear rates showed no significant differences when compared by nominal insert size (small vs. medium vs. large, *p* = 0.563 and *p* = 0.747, respectively) or by component (femoral-to-insert) size ratio (≤1 vs. >1, *p* = 0.885 and *p* = 0.055, respectively). (4) Conclusions: The study found increased volumetric wear in cases of osteolysis alone, with greater wear when combined with clinical loosening compared to other groups. The instability group demonstrated a characteristic peripheral anterior and posterior wear pattern. Insert size and component size ratio seem not to influence wear rates.

## 1. Introduction

Each year, more than one million total knee arthroplasties (TKA) are being performed worldwide, and this number is expected to increase significantly over the next decades [1]. Many patients have seen tremendous benefits from TKA; however, studies have shown that up to 20% of patients are not satisfied with the results of this procedure [2,3]. Consequently, this represents a huge concern to patients, surgeons, implant manufacturers, hospitals, and health care funds [4,5]. The most common indications for revision (or cause for dissatisfaction) in TKA include prosthetic joint infection (PJI), wear of the tibial polyethylene insert, aseptic loosening, periprosthetic fracture, malposition of the implant, mechanical failure, instability, and stiffness of the prosthetic joint [6,7]. There is also a large number of patients with failed TKA, where the exact cause of failure remains unknown. In these cases, a single cause alone would probably not lead to a problem, but a combination of causes could generate severe dissatisfaction, finally leading to revision without a clearly defined diagnosis. The appropriate diagnoses for these knee implants might be extremely difficult to clarify.

Increased wear of the tibial insert and the consequent induced osteolysis and aseptic loosening is the result of a multifactorial problem that involves factors related to the implant, the surgeon, and the patient. Consequently, it may alone account for up to 25% of all TKA revisions [8,9]. Analyses of retrieved inserts allow for a better understanding of TKA failure mechanisms and the detection of factors that cause increased wear [10]. Previous studies with their key findings are presented in Table 1 [10,11,12,13,14,15,16,17,18,19,20,21,22,23,24,25,26,27,28,29,30,31,32]. There is a lack of research on well-defined cohorts of retrieved insert samples, analyzed using modern methods involving information on the 3D wear distribution of the articular surface in different clinical causes for arthroplasty failure.

This implant retrieval study quantitatively assessed insert wear following failed cruciate-retaining (CR) type TKA. To minimize manufacturer-related variables, we analyzed only inserts made from identical gamma vacuum foil (GVF) UHMWPE and designed by a single manufacturer (DePuy Synthes, Johnson & Johnson, Warsaw, IN, USA). Our purpose was to identify (a) whether volumetric wear (adjusted for time in situ) significantly differs among groups of common causes of TKA failure, (b) whether there is a characteristic wear distribution pattern for a common cause of TKA failure, and (c) whether nominal insert size (small vs. medium vs. large) and component size ratio (femur-to-insert) influence linear and volumetric wear rates.

## 2. Materials and Methods

### 2.1. Retrievals

**Figure 1:**
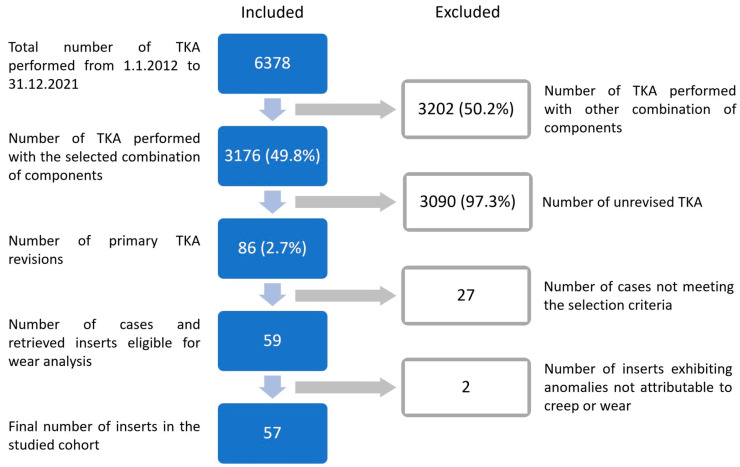
Flow diagram of studied cohort identification. Abbreviation: TKA, total knee arthroplasty.

A prospective cohort of retrieved Sigma^®^ Cruciate-Retaining Curved GVF i2 inserts from DePuy Synthes (Johnson & Johnson, Warsaw, IN, USA), that were removed during the primary revision surgery in Valdoltra Orthopedic Hospital, Slovenia between the years 2013 and 2021, has been included in the IRB approved study. Out of 6378 primary TKAs conducted during this period, 3176 TKAs were implanted with the chosen insert (refer to Figure 1). Among these, 86 cases (2.71%) underwent revision surgery. The current overall number of retrieved TKA components stored in our research department exceeds 600. Retrievals were included in the study if all the following criteria were met: cemented TKA, tibial component Sigma^®^ Modular CoCr Tibia Tray Component (Non-Porous), and femoral component Sigma^®^ CR Femoral Component (Non-Porous). Exclusion criteria comprised missing patient data, absent explant or information on prosthesis type, and lack of surgical report for the index or revision surgery. Out of the 59 retrieved inserts obtained from an equal number of patients meeting the selection criteria, 2 cases exhibited considerably different types of damage, as outlined in the results section. Since these anomalies could not be attributed to creep or wear, they were subsequently excluded from further analysis. Therefore, the final cohort consisted of 57 inserts. Clinical and demographic data of patients were obtained from the institutional Valdoltra Arthroplasty Registry [33] and electronic clinical records. The study has been registered with ClinicalTrials.gov (ID NCT05482438).

### 2.2. Scanning Process and Insert Wear Analysis

**Figure 2:**
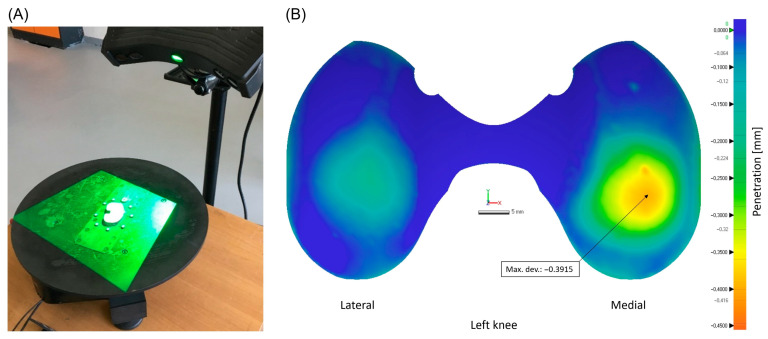
(**A**) Process of metrology data collection from an insert with an optical scanner. (**B**) Example of a penetration wear map of the articular load-bearing surface with a point of maximal linear wear indicated with an arrow. Abbreviations: Max. dev., maximal deviation.

After the initial cleaning, a white anti-glare coating agent (3D SCAN-IT IP-25 by Samson Kamnik d.o.o., Kamnik, Slovenia) was uniformly applied with a spray gun on the surface of the insert. Then, it was placed on a rotating platform and scanned using an EviXscan 3D Heavy Duty Quadro 3D optical scanner (EVIXSCAN 3D, Bielsko-Biala, Poland) from multiple angles. For point cloud processing, we utilized the measurement software EviXscan 3D Suite 2.7 (EVIXSCAN 3D, Bielsko-Biala, Poland). With the described systems, we acquired a stereolithographic (STL) file of each retrieved insert. Geomagic Control X 2022.1.0 software (3D Systems, Rock Hill, SC, USA) was utilized for post-processing the measurements and conducting a comparison between the scanned insert with its reference computer-aided design (CAD) model of appropriate dimensions provided by DePuy Synthes (DePuy International Ltd., Leeds, UK). The superimposition of the scanned file with the corresponding CAD model for wear calculation was performed based on the best-fit alignment process, aligning with both plain nonbearing articular and lateral surfaces to ensure optimal accuracy. Nonbearing articular parts were subsequently removed perpendicularly, to allow for more precise further measurements and relevant presentations of the articular load-bearing surface, including the creation of a linear penetration wear map (Figure 2).

We calculated the linear wear of the articular load-bearing surface, defined as the maximum deviation in millimeters from the reference at any point of this surface. Also, the volumetric wear of the same surface, measured in cubic millimeters, was determined by subtracting the volume of the retrieved insert from the reference CAD model. In simple terms, linear wear refers to the maximum depth of material loss, while volumetric wear represents the total amount of material lost. The linear and volumetric wear rates were calculated by dividing the wear by the component time in situ (time from primary TKA to revision operation), reported as mm/year and mm^3^/year, respectively.

### 2.3. Determination of Characteristic Wear Patterns

**Figure 3:**
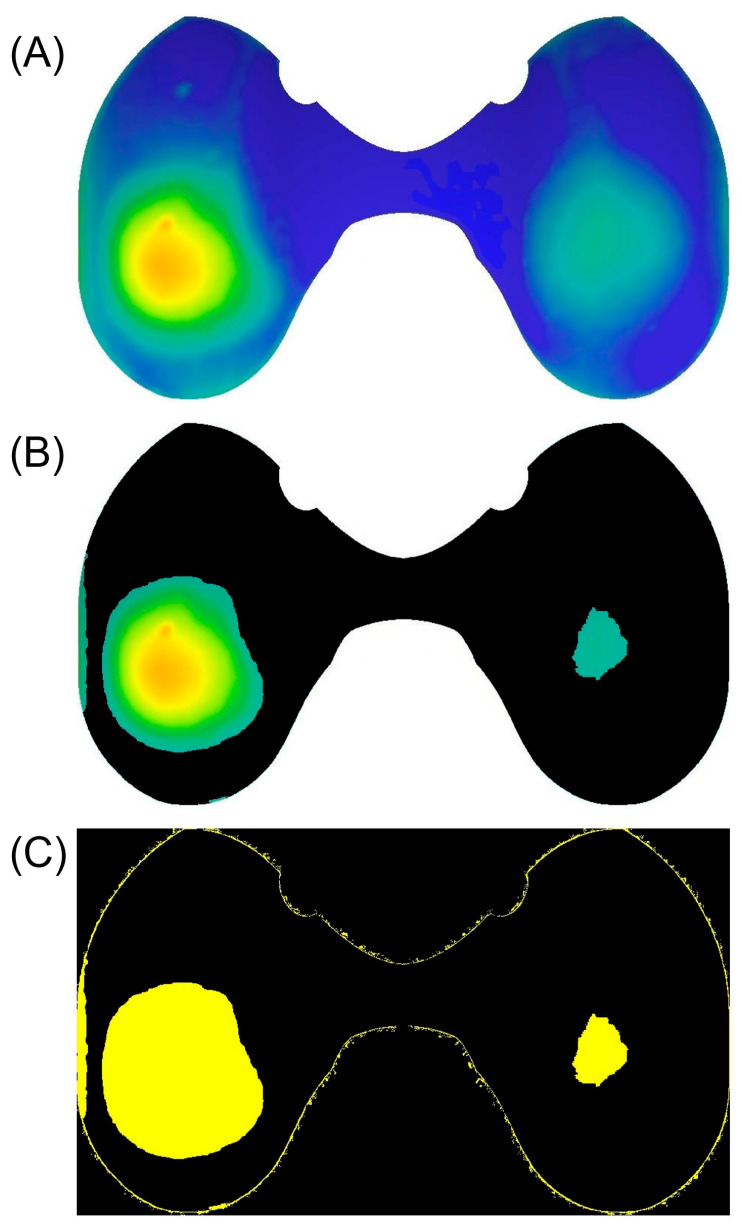
Image processing steps of a penetration wear map before superimposition with other images within the group. (**A**) Cropped and horizontally mirrored image (inserts of left knees only). (**B**) Areas with no or less damage removed (shown in black); more damaged areas with depth still visible. (**C**) Binarized more damaged areas represented in yellow.

Linear penetration wear maps of inserts from the respective groups associated with common causes of TKA failure were further analyzed to identify potential wear distribution patterns. Image processing was conducted using Python 3.11.2 (Python Software Foundation, Wilmington, DE, USA) and OpenCV (Palo Alto, CA, USA) libraries, with a primary focus on generating an “average image” of each group. In this process, each image contributed a proportional share to the final result. The substantial challenge encountered was the variability in the size and shape of the inserts. To ensure precise superimposition, insert image sizes were normalized, and the side of the insert was also normalized (left inserts were horizontally mirrored) to capture potential medial vs. lateral characteristics. Point cloud data from measurements were converted into heat maps, which were then used to create the average image. To enhance the visibility of areas with considerable wear and exclude those with less or no wear, initial penetration wear maps were converted into binary images based on the threshold determined by the actual result analysis (Figure 3). This method disregarded wear depth information, concentrating solely on the locations of considerable wear. Ultimately, processed images were superimposed to create a single average binary image, thereby enhancing visibility and highlighting a potential wear distribution pattern within a group.

### 2.4. Identification of All Possible Causes for TKA Failure

In addition to examining the cause of revision surgery indicated by the operating surgeon, we conducted a review of each case, searching for any potential additional causes contributing to TKA failure. To accomplish this, we reviewed perioperative and follow-up records, along with the available radiological, laboratory, and microbiological studies. Ultimately, each case was categorized into one or more of the following groups representing the most common causes of TKA failure, using validated guidelines:

1. Confirmed PJI according to the current European Bone and Joint Infection Society (EBJIS) definition [34];
2. Osteolysis (defined as progressive radiolucent lines one year after index TKA with radiological assessment according to the Knee Society Roentgenographic Evaluation and Scoring System) [35,36];
3. Clinical loosening of the component (intraoperative assessment—evident movement of a prosthetic component within the bone);
4. Joint malalignment or component malposition (in the coronal plane: deviation of hip-knee-ankle angle (HKAA) from 180 ± 3° (varus/valgus malalignment), deviation of mechanical lateral distal femoral angle (mLDFA) from 90 ± 3°, deviation of medial proximal tibial angle (MPTA) from 90° to 87°, in the sagittal plane, deviation of distal femoral flexion angle (DFFA) from 90° to 87° and deviation of tibial slope (TS) from 0° to 7°) [37];
5. Instability (positive history and clinical examination of abnormal and excessive displacement of a knee prosthesis [38] with intraoperative finding or positive stress radiography);
6. Other isolated causes (joint stiffness—flexion contracture greater than 10° and/or flexion limit ranging from 90° [39] and/or pain not classified in any of the previous groups).

### 2.5. Determination of Groups Based on Nominal Insert Size and Component Size Ratio

All total knee prostheses were implanted following the manufacturer’s instructions and the stated nominal size compatibility of the components.

Based on the median nominal insert size value of 3, we formed the following three groups: small (sizes 2 and 2.5), medium (size 3), and large (sizes 4 and 5).

Based on the nominal component size ratio (femoral-to-insert; F:I ratio) of each case in the cohort, we established two groups. The first group included cases with F:I ratios ≤ 1, whereas the second group included cases with F:I ratio > 1. It is important to mention that we treated the size of the femoral components 3 and 4 N as equivalent, due to their identical mediolateral dimensions. Consequently, the F:I ratios of femoral component sizes 3 and 4 N to insert size 3 were both 1.

### 2.6. Radiological Assessment

**Figure 4:**
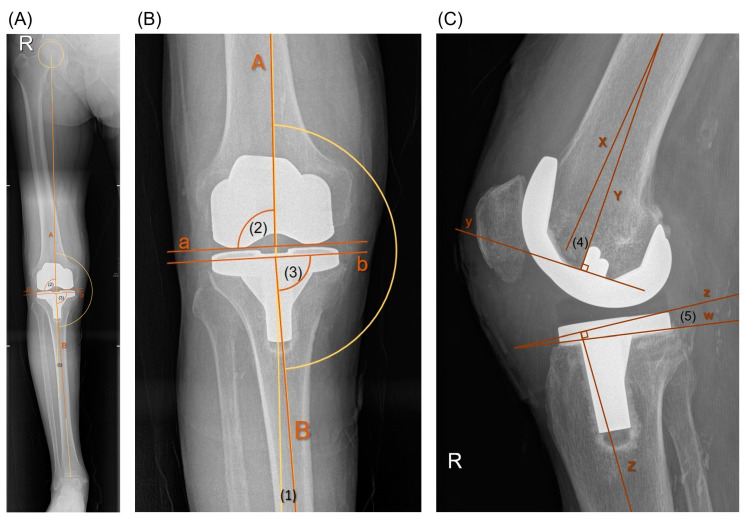
(**A**) Long-leg standing AP view. (**B**) Enlarged image (**A**): A = femoral mechanical axis; B = tibial mechanical axis; a = distal horizontal tangent to the femoral component; b = proximal horizontal tangent to the tibial component; (1) hip-knee-ankle (HKA) angle; (2) mechanical lateral distal femoral angle (mLDFA) = angle within [A] and [a]; (3) medial proximal tibial angle (MPTA) = angle within [B] and [b]. (**C**) Lateral knee view: X = femoral anatomical axis in sagittal plane; y = horizontal tangent to the femoral component; Y = perpendicular axis to [x]; (4) distal femoral flexion angle (DFFA) = angle within [X] and [Y]; Z = tibial anatomical axis in sagittal plane; z = perpendicular horizontal to Z; w = proximal tangent to the tibial component; (5) tibial slope (TS) = angle within [w] and [z].

Radiological diagnostic studies were conducted using the Enterprise Imaging 8.2.0.140 software (AGFA HealthCare, 2640 Mortsel, Belgium) on long-leg standing anteroposterior (AP) and standard short film lateral knee view [37]. Alignment and component positioning were assessed based on HKAA, mLDFA, MPTA, DFFA, and TS (refer to Figure 4).

### 2.7. Microscopic Analysis of Retrieved Inserts

Selected inserts were analyzed for visual wear patterns and damage using a digital microscope (ZEISS Smartzoom 5, Carl Zeiss AG, Oberkochen, Baden-Württemberg, Germany) and a scanning electron microscope (SEM) (Thermo Fisher Quanta 650 ESEM, Thermo Fisher Scientific Inc., Waltham, MA, USA). For SEM analysis, an ETD (Everhart-Thornley detector) was used in secondary electron (SE) mode with an acceleration voltage of 10 kV. Before the analysis, inserts were coated with a 15 nm thick carbon layer to prevent charging and improve imaging.

### 2.8. Statistical Analysis

Categorical variables were summarized using frequencies and percentages, while continuous variables were summarized using means and standard deviation (SD) or, in cases of non-normality as tested by the Shapiro–Wilk test, using the median and interquartile range (IQR). Pearson’s correlational coefficient (R) with 95% confidence interval (CI) was used to estimate the association between linear or volumetric wear and time in situ. The associations between volumetric wear and causes for TKA failure were estimated using a multiple linear regression model adjusted for time in situ. Osteolysis and clinical loosening of the component as causes of TKA failure were merged into one variable with the following categories: both causes present, osteolysis alone (indicating osteolysis without clinical component loosening), and none of them. The other causes of TKA failure were included in a regression model as dichotomous (yes vs. no) variables. The differences in linear and volumetric wear rates according to nominal insert size (small vs. medium vs. large) and component size ratio (≤1 vs. >1) were tested using the Kruskal–Wallis test and the Mann–Whitney test, respectively. A *p*-value < 0.05 was considered statistically significant. Analysis was performed using R 4.3.2 (Foundation for Statistical Computing, Vienna, Austria) statistical software.

## 3. Results

### 3.1. Patient Demographics, Insert Characteristics and Wear Correlation with Time In Situ

Articular load-bearing surface wears were computed for 57 inserts. The median scanning error was 9 (IQR 8–9) μm. The mean age of patients was 69.6 years and 57.9% were women. The median time in situ of inserts was 17.4 months. The median linear and volumetric wear were 0.18 mm and 63.7 mm^3^, respectively. Overall, 21.1% of inserts were categorized as small, 36.8% as medium and 42.1% as large. Moreover, 54.4% of inserts were categorized by nominal component size ratio as ≤1, while 45.6% were categorized as >1. Patient demographics and insert characteristics are presented in Table 2.

**Figure 5:**
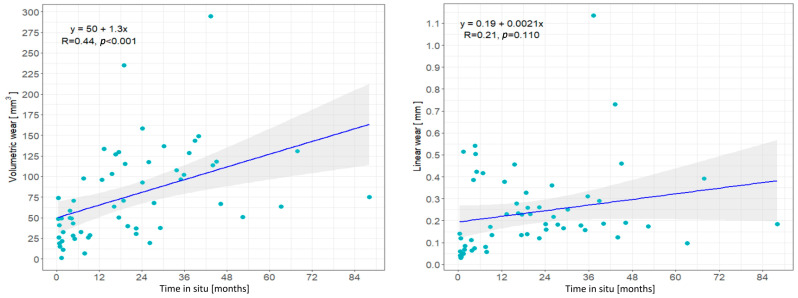
Illustration of the relationship between volumetric wear (**left**) and linear wear (**right**) with time in situ deduced using the linear regression model with regression lines (dark blue) and 95% confidence intervals (grey area). The light blue dots represent individual cases.

The relationship between linear wear and volumetric wear with time in situ is shown in Figure 5. There was a statistically significant correlation between volumetric wear and time in situ (R = 0.44, 95% CI 0.27–0.64, *p* < 0.001). The linear regression model demonstrated that the estimated initial volume loss at time 0 (intercept) was 50 mm^3^ (95% CI 30.3–69.8 mm^3^) and an average increase (slope) of 1.3 mm^3^ per month or 15.4 mm^3^ per year (95% CI 7–23.9 mm^3^), representing the volumetric wear rate. In contrast, the linear wear and time in situ were not significantly correlated (R = 0.21, 95% CI −0.03 to 0.43, *p* = 0.110). Additionally, the analysis of outliers was performed, and the results remained unchanged regarding statistical significance.

### 3.2. Distribution of TKA Failure Causes and Their Association with Volumetric Wear

**Figure 6:**
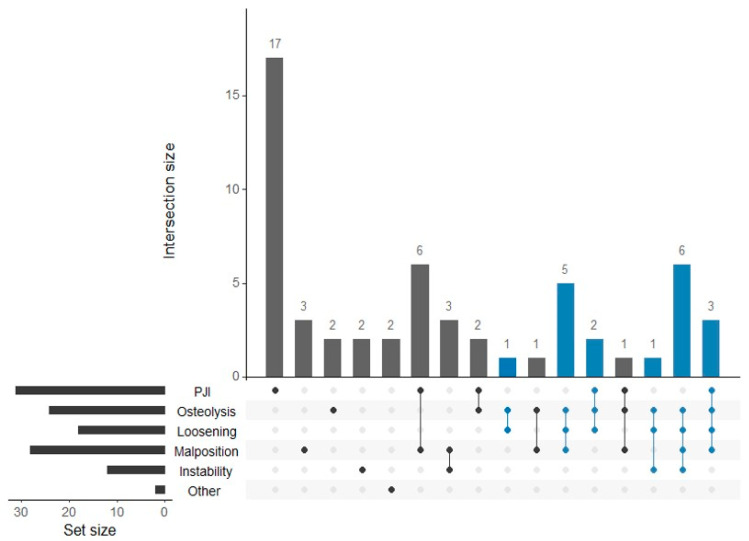
The attribute plot of identified possible causes of TKA failure. Abbreviations: loosening, clinical loosening of the component; malposition, joint malalignment or component malposition; other, other isolated causes; PJI, confirmed prosthetic joint infection. Note: Cases with identified osteolysis and clinical loosening of the component are shown in blue.

After the identification of all possible causes of TKA failure within their respective groups, the following distribution was observed: 31 (54.3% out of 57) cases of confirmed PJI of which 11 (19.2%) were classified as early, 24 (42.1%) cases of osteolysis of which 6 (10.5%) cases were without concomitant component loosening, 18 (31.6%) cases of clinical loosening of the component (of which 13 cases or 72.2% were aseptic), 28 (49.1%) cases of joint malalignment or component malposition, 12 (21.1%) cases of instability (of which 10 cases or 83.3%) were recognized as mediolateral due to insufficiency of collateral ligaments), and 2 (3.5%) cases of other isolated causes (including instances of joint stiffness and patellofemoral pain). Due to the limited number of cases within the last group, it was underpowered for statistical analysis and thus excluded from further study. In 26 (45.6%) cases, a single detected cause of TKA failure was identified, while the rest exhibited multiple possible causes (2 in 13 (22.8%), 3 in 9 (15.8%) and 4 in 9 (15.8%) cases). The majority of single-cause cases were within the group of confirmed PJI (54.8%) (refer to Table 3 and Figure 6).

Notably, osteolysis was observed in all cases of clinical loosening of the component, while joint malalignment or component malposition was identified in 77.8% of cases within this group. Hence, the most common combination of causes of TKA failure was osteolysis with clinical loosening of the component and joint malalignment or component malposition (24.6%). Joint malalignment or component malposition was detected in 75.0% of cases of instability, whereas instability was identified in 32.1% of cases of joint malalignment or component malposition. Cases of confirmed PJI most commonly displayed concomitant joint malalignment or component malposition, observed in 32.3% of cases. In 79.2% of cases with varus or valgus malalignment, the location of maximum linear wear corresponded to the medial or lateral part of the insert, respectively.

The multivariate regression model with volumetric wear as the dependent variable showed a statistically significant association with variables time in situ (*p* = 0.013) and osteolysis with or without clinical loosening of a component (Table 4). Patients with identified osteolysis alone and those also with clinical loosening of the component had significantly higher volumetric wear when compared to those without both causes (*p* = 0.016 and *p* = 0.009, respectively). All other causes were not significantly associated with volumetric wear.

### 3.3. Wear Distribution Patterns

**Figure 7:**
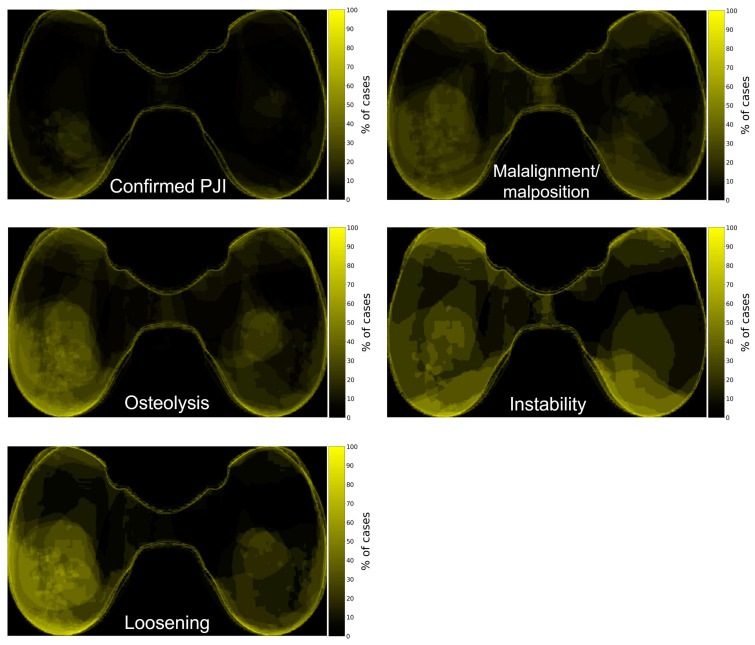
Wear distribution within groups of causes of TKA failure. In the instability group, areas of higher frequency of wear are located in the peripheral anterior and posterior areas of the articular load-bearing surface.

The threshold for binary image creation was determined to be 0.13 mm. To visualize wear patterns, an average binary image was created for each group of possible causes of TKA failure in a heatmap style. In this representation, the brightest areas indicate the highest frequency of considerable wear, whereas the darkest areas signify the lowest frequency of considerable wear in specific areas of the articular load-bearing surface. All inserts were normalized to the right knee, thus referring to the left part of the representation as medial and the right part as lateral. The results from the instability group differentiated from the others with a peripheral antero-posterior wear distribution (refer to Figure 7). In other groups, a characteristic pattern was not observed.

### 3.4. Comparison of Wear Rates Based on Insert Size and Component Size Ratio

Linear and volumetric wear rates were also compared based on the nominal insert size and component size ratio (femoral-to-insert) using the Kruskal–Wallis test and the Mann–Whitney test, respectively. The differences were not significant (nominal insert size: small vs. medium vs. large, *p* = 0.563 and *p* = 0.747, respectively; nominal component size ratio: ≤1 vs. >1, *p* = 0.885 and *p* = 0.055, respectively).

### 3.5. Microscopic Wear Patterns and Presentation of Excluded Inserts

**Figure 8:**
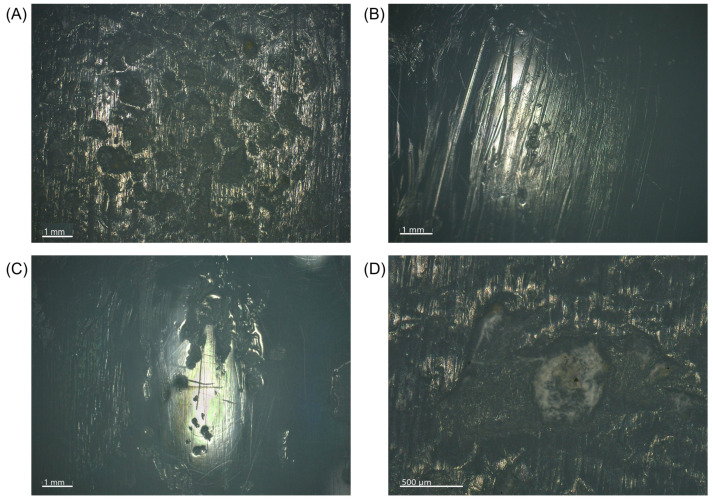
Microscopic images showing the predominant visual wear patterns within the cohort. (**A**) Pitting. (**B**) Scratching. (**C**) Burnishing. (**D**) Embedded debris at the bottom of a pit.

Microscopic analysis of the articular load-bearing surfaces within our cohort demonstrated mainly abrasive and adhesive types of visual wear damage patterns, consisting of a combination of pitting, scratching, and burnishing. Only in individual cases, have we also noticed some embedded debris at the bottom of pits, that may represent bone cement Figure 8).

**Figure 9:**
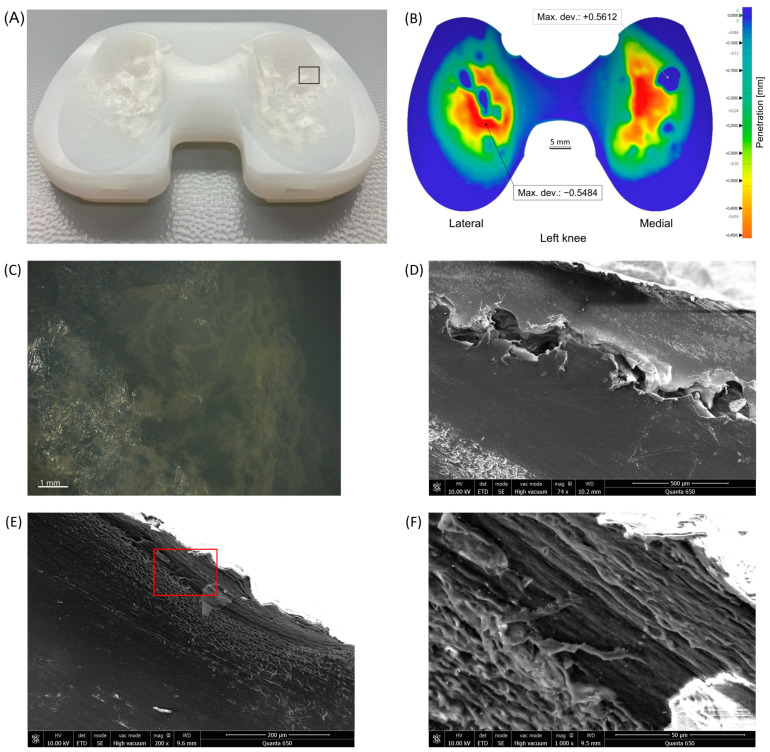
Insert with articular load-bearing surface protuberances. (**A**) Photo. (**B**) Penetration wear map with resulting volumetric wear of −72.46 mm^3^ (note; a negative value indicates an increase in volume). (**C**) Microscopic view of the black rectangle shown in (**A**) exhibiting discoloration and layer separations observed using a digital microscope. (**D**) Scanning electron microscope image of a section through a protuberance showing rough layer separation. (**E**) Scanning electron microscope image of a section through an area adjacent to a protuberance showing delamination. (**F**) Enlarged view of the red rectangle shown in (**E**). Abbreviations: det, detector; ETD, Everhart-Thornley detector; HV, high voltage; mag, magnification; SE, secondary electrons; vac, vacuum; WD, working distance.

In two cases, considerably different types of damage were observed over the load-bearing areas, rendering appropriate volumetric wear calculations unfeasible; therefore, these two inserts were excluded from the analysis. In the first case, revision surgery was performed after 8.3 years in situ due to painful aseptic loosening of the tibial component and progressive joint varus alignment. The retrieved insert exhibited multiple protuberances on the central part of the articular load-bearing surfaces. Microscopic analysis revealed characteristic oxidation-related fatigue damage patterns, including layer separation and delamination (Figure 9).

**Figure 10:**
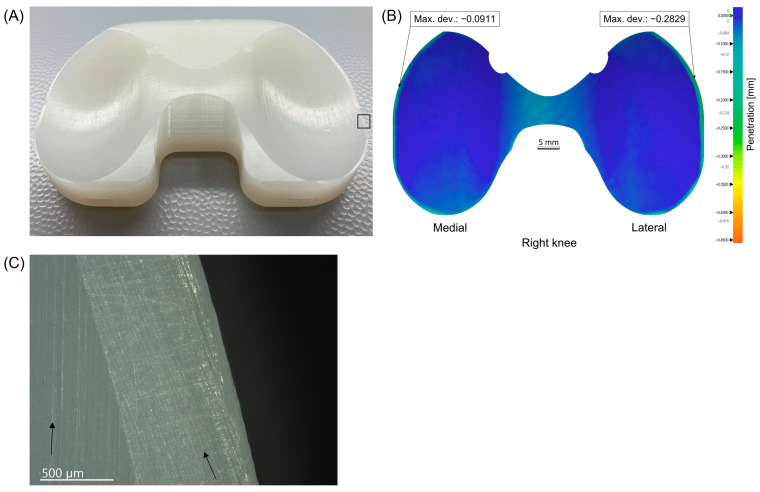
Insert showing a significant discrepancy from its reference CAD model. (**A**) Photo. (**B**) Penetration wear map. (**C**) Microscopic view of the black rectangle shown in (**A**), with arrows indicating the direction of parallel machining marks.

In the second case, a notable discrepancy was observed across the entire edge and intercondylar portion of the load-bearing surface between the insert and its reference CAD model. This deviation is attributable to manufacturing reasons, given the very short time in situ of only 8 days and the revision surgery due to subcutaneous seroma (Figure 10).

## 4. Discussion

Our objectives were to determine whether volumetric wear (adjusted for time in situ) differs among groups of common causes of TKA failure, whether a group exhibits a characteristic wear distribution pattern, and also to investigate whether nominal insert size (small vs. medium vs. large) and component size ratio (femur-to-insert) influence linear and volumetric wear rates. The results of this study suggest that increased volumetric wear can be expected in cases with identified osteolysis alone and in combination with clinical loosening of a component. A characteristic combined peripheral anterior and posterior wear pattern has been discovered in instability cases. Lastly, nominal insert size and component size ratio seem not to influence linear and volumetric wear rates.

Increased wear or wear rates have been observed with longer time in vivo [31] and in cases where PE wear (including osteolysis) and loosening/subsidence were reported as reasons for revision [10], which is consistent with our findings. It is important to emphasize that component loosening rarely occurs without previous osteolysis, as osteolysis in the long term leads to bone loss, and finally the loosening of implants [40]. Consequently, we identified osteolysis in all of our cases with clinical loosening of a component. Highly cross-linked polyethylene (HXLPE), with its enhanced wear resistance, may theoretically be a better option for individuals with increased wear risk factors, such as male gender and younger age [10]. However, studies have shown that HXLPE did not improve clinical and radiographic outcomes after TKA [41,42,43]. Concerns remain regarding its mechanical properties compared to conventional UHMWPE, including reduced toughness, ductility, and fatigue resistance, making its benefits still controversial.

To our knowledge, a characteristic wear distribution pattern specific to a particular cause of failure has not yet been described. Interestingly, the instability-related peripheral anterior and posterior wear pattern appeared, even though the reported clinical instability was mediolateral in over 80% of the cases within the group. This could be explained by the increased valgus-varus laxity converting to an anteroposterior instability, leading to increased creep or wear in the anterior-posterior parts of the insert. Overall, the most prevalent wear pattern observed within our cohort exhibits a bias toward medial wear, which aligns with previous studies [16,32]. This can be attributed to the predominant varus knee malalignment (57.1% compared to 25.0% of valgus knees) within the group of joint malalignment or component malposition. Additionally, nearly 90% of cases in this group were also part of at least one other group of the most common causes of TKA failure, further contributing to these results. Monitoring patients with early signs of osteolysis and/or instability allows for timely interventions, potentially improving long-term outcomes.

There is a lack of information on whether insert size and component size ratios influence articular-side wear. In our study, neither feature appeared to be significant to the extent of wear. On the other hand, in a similar study, backside wear scores were significantly higher in medium compared to large insert sizes [31]. Factors like patient weight, activity level, or other biomechanical conditions could interact with component size in ways not fully reflected in our data. For example, larger components may behave differently under extreme conditions, such as in heavier or more active patients. Future studies could explore these potential interactions by examining larger cohorts and assessing how these variables collectively impact wear rates.

Describing wear in terms of dimensional change may encompass the plastic deformation (creep or bedding-in) of the PE bearings [44] in calculating wear (material loss) [45]. However, volumetric wear measurements provide a more accurate representation of material loss compared to qualitative surface damage appearance [26]. Moreover, this measurement is a key determinant of the number of polyethylene (PE) particles generated, which contribute to the osteolytic process. It is important to mention that third-body debris (bone, bone cement, and metal particles) remains a contributor to total wear through abrasive wear mechanisms [46,47].

Due to the inclusion of inserts failing with short time in situ, calculating wear rates (per year) would be overestimated [48]. Nonetheless, comparisons of volumetric wear between groups were adjusted for time in situ. Considerable volume loss was observed in inserts with a short time in situ, most probably attributed to creep. Simulator tests have demonstrated that the majority of creep occurs during the initial millions of cycles [49,50]. The estimated average increase in volume loss or wear of 15.4 mm^3^ per year, as determined by a linear regression model, was similar to previously published results [25,26,31].

Our analysis indicates that, particularly in aseptic cases, more causes in conjunction lead to unsuccessful TKA and eventual revision surgery. Consequently, we advise arthroplasty registries to report all of them. This would give a more detailed insight into the multifactorial process of TKA failure.

Study strengths include a completely homogeneous cohort of implants comprising a single type of UMHWPE insert and set of combined components (including wear-decreasing polished tibial tray surface [19]), along with a retrospective analysis of prospectively collected data sourced from an established institutional register of arthroplasty [33]. Each case of failure has been thoroughly reviewed for potential additional factors contributing to TKA failure. Long-leg standing radiographs enabled the assessment of medial/lateral wear distribution in cases of varus/valgus malalignment. Three-dimensional optical scanning has proven a simple, rapid, and precise method for analyzing wear in retrieved inserts, ensuring the reliable reconstruction and presentation of a penetration wear map [51,52]. In our study, wear was analyzed only on the insert load-bearing areas. This approach minimizes the potential bias in volume loss that may arise from damage to other articular areas, such as deformations produced during insert extraction. However, these damages on the analyzed surface may have still influenced our results, particularly regarding linear wear, which may explain the unexpected absence of correlation with time in situ [10,32]. This damage can sometimes be more obvious, particularly in cases with a short time in situ. However, due to the general uncertainty of the etiology of these changes, all maximal linear wear points on the articular load-bearing surface were considered. Consequently, it seems plausible that our data showed no statistically significant correlation between linear wear and time in situ due to a 0-time intercept of approximately 200 μm and no lower.

On the other hand, our study has several limitations. As a retrospective analysis of implant retrievals, there is an inherent risk of selection bias, particularly since the types of implants retrieved may not be representative of the broader population of well-functioning devices in use. Furthermore, the variability in patient-specific factors, such as activity level, and individual biomechanics, may influence wear and cannot be fully controlled in this study. We lacked information regarding the level of physical activity among patients after primary TKA implantation. However, a recent meta-analysis found no association between high physical activity levels and an increased risk of revision surgery during the first twelve years post-TKA [53]. The analysis in this study was limited to the articular surface. Backside wear could not be properly analyzed with the scanning technique because the insert locking tabs, damaged by extraction, often did not allow the scanning of covered parts. A recent simulator study involving similar conventional UHMWPE CR-type inserts estimated that backside damage contributes to up to 20% of the total wear of the insert [54]. However, it should be noted that the authors analyzed inserts with central dovetail locking mechanisms mounted on non-polished tibial trays, two factors previously identified to increase the extent of backside wear [28]. While our cohort size was relatively small, it is consistent with previous research focused on a single PE insert [19,23,26,31]. Findings may not be generalizable as only one implant design was investigated. Nonetheless, the results could potentially extend to other CR TKA designs. During the radiological assessment, we also examined for potential rotational malposition of components on CT scans [55]. However, this investigation was conducted only in a limited number of cases. We were unable to obtain information regarding the manufacturing tolerance, which refers to the dimensional variability between the CAD model and the real insert after manufacturing. As presented, such discrepancies could have significant implications when utilizing our method or a similar approach for wear analysis, particularly when relying on CAD models or unworn, new inserts [22] as references. An optimal superimposition of images could not be achieved because of the variability in the shape of articular load-bearing areas among different insert sizes, including thicknesses. Nevertheless, significant marginal damage remained visible.

A novel aspect of this study involves the detection methodology and presentation of wear distribution patterns per identified cause of TKA failure. This significant 3D upgrade to established quantitative retrieval polyethylene analysis provides a deeper insight into TKA wear mechanisms in relation to clinical circumstances. Moreover, it has the potential to aid in diagnostically demanding cases and to guide future improvements in implant designs.

## 5. Conclusions

Our findings indicate a significant increase in volumetric wear in cases where osteolysis was present alone, with an even greater increase observed when it was combined with clinical loosening of the component, compared to other common causes of TKA failure. A characteristic peripheral anterior and posterior wear pattern has been shown in the instability group. Reporting all contributing causes in arthroplasty registries is essential for gaining a comprehensive understanding of the multifactorial nature of TKA failure, especially in aseptic cases.

## Figures and Tables

**Table 1:** Studies analyzing the wear of retrieved TKA polyethylene inserts.

Study, Year	Insert Type	Implant	Nu.	Time In Situ (Months)	Assessment/Measuring Method	Wear Rate	Notes/Key Findings
Li et al. [11], 2002	FB	Anatomic Modular Knee (AMK)^®^, Genesis^®^, Insall-Burstein II^®^, PFC^®^	55	(1–73)	Visual assessment of wear patterns, CMM	NA	Backside PE wear results as a significant contributor to the generation of PE debris.
Conditt et al. [12], 2005	CR	AMK^®^	15	(36–146)	Surface laser profilometry	138 ± 95 mm^3^/year *	The predicted volume PE loss because of backside wear is substantial and may be sufficient to induce osteolysis.
Ho et al. [13], 2007	MB, FB	New Jersey LCS^®^ RP, Miller Galante I^®^ FB	51	115 (48–162)	Visual assessment of wear patterns	NA	Low-grade wear (burnishing, abrasion, and cold flow) more common in MB knees; high-grade wear (scratching, pitting, metal embedding, and delamination) more common in FB knees.
Garcia et al. [14], 2009	MB	LCS^®^ RP, Sigma^®^ RP	40	30 (0.3–191)	Visual assessment of wear patterns	NA	No significant difference between medial and lateral wear. Wear increases with time in situ, patients’ weight, and BMI. Increased wear in unstable knees.
Lu et al. [15], 2010	MB, FB	LCS^®^, PCA^®^, Miller-Galante^®^	73	121 (48–162)	Visual assessment of wear patterns	NA	Increased articular surface wear in FB knees. Increased backside wear in MB knees. Wear in MB knees not associated with time in situ and BMI.
Srivastava et al. [16], 2011	CR	PFC^®^	16	92.4 (12–156)	Visual assessment of wear patterns, Laser scanner CMM	131 ± 78 mm^3^/year	Varus malalignment of the tibial component (≥3°) increases medial compartment wear and overall insert wear even when the knee is in neutral alignment.
Knowlton et al. [17], 2012	CR	Miller-Galante II^®^	9	(4.1–30.2)	Laser scanner CMM	39.2 ± 7.2 mm^3^/year	An autonomous mathematical reconstruction can be used to effectively measure volume loss in retrieved tibial inserts.
Wimmer et al. [18], 2012	CCK/PS, CR	Insall-Burstein II^®^, Miller-Galante II^®^	69	18.6 (0.8–59.7), 19.9 (0.4–64.3)	Visual assessment of wear patterns	NA	More conformity (CCK/PS) increase surface fatigue damage in TKA compared to less conform designs (CR).
Berry et al. [19], 2012	MB, FB	^a^ Sigma^®^ RP, ^b^ Sigma^®^ with Ti or CoCr trays	312	^a^ 36 (0.4–124), ^b^ 72 (2–179)	Insert thicknesses measurements using a dial indicator	^a^ 0.04 mm/year; ^b^ 0.07 mm/year (total); ^b^ 0.02 mm/year *; ^b^ 44 mm^3^/year *	Wear rate lower in RP compared to FB inserts. Backside wear rate lower for FB inserts mated with CoCr trays than for rough Ti trays. Inserts against polished trays (RP or FB) showed no increase in wear rate over time. The wear rate of PS and CR inserts was not different.
Stoner et al. [20], 2013	MB, FB	PFC^®^ Sigma RP, PFC^®^ Sigma	42	24 (5–46) 65 (6–134)	Visual assessment of wear patterns	NA	The increased total damage score on the RP, coupled with increased surface area damaged and the propensity for third-body debris, indicates no damage advantage to MB design.
Paterson et al. [21], 2013	HF, PS	Genesis^®^ II	40	20 (4–43)	Visual assessment of wear patterns, micro-CT	NA	Increased wear of post and backside surface of HF inserts due to ability of higher flexion of the knee.
Engh et al. [22], 2013	MB, FB	LCS^®^, Sigma^®^ RP, PFC^®^ Sigma	24	52	Volumetric wear analysis with micro-CT	43 ± 25 mm^3^/year; 74 ± 49 mm^3^/year	Micro-CT can determine the volume and location of wear in retrieved inserts. Significant influence of manufacturer tolerance when short follow-up or low wear present. A more accurate method for greater wear and simulator studies when repeated measurements possible.
Pang et al. [23], 2014	PS	Genesis II^®^	83	42 (4–124)	Visual assessment of wear patterns	NA	Limb malalignment (varus) and JLE (>5 mm) resulted in more wear. In cases with JLE, an external rotation subluxation wear pattern was found with more damage over the posteromedial aspect of the post.
Holleyman et al. [24], 2014	FB	many different	30	156 (36–240)	Profilometry with a non-contact profilometer	NA	Surface roughness and skewness measured on the insert undersurface and the tibial baseplate of explants. Decreased backside wear found when a polished tibial tray was used compared to an unpolished design.
Schwarzkopf et al. [25], 2015	CR	PFC^®^	70	142 (15–289)	Insert thicknesses measurements	^a^ 0.0063 mm/year *; 14.2 mm^3^/year *; ^b^ 0.05 mm/year *; 117 mm^3^/year *	More conforming ^b^ (curved) tibial inserts demonstrated more backside-normalized wear than the flatter ^a^ (posterior lipped) inserts.
Knowlton et al. [26], 2017	CR	Miller-Galante^®^ II	64	36 (0.4–108)	Visual assessment of wear patterns, video microscopy, low-incidence laser CMM	12.9 ± 5.97 mm^3^/year; 0.035 ± 0.017 mm/year medially; 0.034 ± 0.011 mm/year laterally	Although striated patterns were the main contributors to volume loss, visual damage patterns were only moderate predictors. Geometric volume loss provides a more accurate quantification of in vivo wear.
Li et al. [27], 2017	PS, CCK	many different	156	>24	Visual assessment of wear patterns	NA	Higher damage in TKAs with postoperative varus alignment.
Łapaj et al. [28], 2017	CR, PS	many different	102	30	Visual assessment of wear patterns	NA	A smooth tibial tray surface and a peripheral locking mechanism reduce backside wear in vivo. No significant differences were found between damage scores in CR vs PS inlays or between genders.
Sisko et al. [29], 2017	CR, PS	AMK^®^, Sigma^®^, Scorpio^®^, Triathlon^®^, Genesis^®^ II	30	>48	Visual assessment of wear patterns, linear wear measurement with micro-CT	Polished designs 0.0102 ± 0.0044 mm/year *; Non-polished designs 0.0224 ± 0.0119 mm/year *	Total backside damage scores and linear wear rates were highest, involving the non-polished design with only a peripheral rim capture.
Affatato et al. [30], 2020	CR, PS	many different	12	70 (12–139)	Visual assessment of wear patterns, topographical analysis	NA	Damage patterns consistent with respect to the main prosthetic components movements. No significant difference found between surface roughness measurements, patient BMI, age at revision, and time in situ.
Pourzal et al. [31], 2020	CR	NexGen^®^	59	61.2	Laser scanner CMM	11.6 mm^3^/year	Increased wear rates with JLE and increased PTS (>7° vs. <3°). Increased backside wear scores of medium compared to large insert size.
Tone et al. [32], 2020	UC, PS	Inserts form B. Braun Aesculap (Tuttlingen, Germany)	13	^a^ (0.75–8.5); ^b^ (15.6–97.2)	Raman spectroscopy	^a^ 0.055 ± 0.020 mm/year on med. LD; ^b^ 0.041 ± 0.020 mm/year on lat. LD	A strong correlation between the amount of wear and time in situ. Increased insert thickness reduction in medial ^a^ compared to lateral ^b^ load zone.
Currier et al. [10], 2021	CR, PS	Sigma^®^, Sigma^®^ RP, NexGen^®^, Triathlon^®^, Genesis^®^ II	1585	58 (0–290)	Calculation of wear based on the reference thickness of inserts with survival <16 months	from 0.021 mm/year (PJI group) to 0.067 mm/year (PE wear group)	Wear rate increased with duration in vivo. Lower wear rates associated with older patients, females, polished modular tibial tray surfaces, HXLPE, and constrained TKA designs.

Note: Time in situ and wear rates are expressed as medians with range or standard deviation. The asterisk (*) after a value refers to the backside wear rate. Abbreviations: BMI, body mass index; CCK, constrained condylar knee; CMM, coordinate measuring machine; CR, cruciate retaining; FB, fixed-bearing; HF, high flexion; HXLPE, highly cross-linked polyethylene; JLE, joint line elevation; lat., lateral; LD, load zone; MB, mobile bearing; med., medial; NA, not available; Nu., number; PE, polyethylene; PJI, prosthetic joint infection; PS, posterior stabilized; PTS, posterior tibial slope; RP, rotating-platform; TKA, total knee arthroplasty; UC, ultra-congruent.

**Table 2:** Patient demographics and insert characteristics.

	n = 57
Gender, female; n (%)	33 (57.9%)
Age, years; mean ± SD	69.6 ± 7.8
BMI; mean ± SD	32.2 ± 5.1
Time in situ, months; median (IQR)	17.4 (4.4–32.0)
Linear wear, mm; median (IQR)	0.18 (0.11–0.32)
Volumetric wear, mm^3^; median (IQR)	63.7 (32.6–114.1)
TKA side, right; n (%)	29 (50.9%)
Insert size; n (%)	
small	12 (21.1%)
medium	21 (36.8%)
large	24 (42.1%)
Nominal component size ratio (F:I), n (%)	
≤1	31 (54.4%)
>1	26 (45.6%)

Abbreviations: n, number of inserts; SD, standard deviation; BMI, body mass index; IQR, interquartile range; F:I, femoral-to-insert.

**Table 3:** Number (%) of cases per number of identified possible causes of TKA failure.

	All	Confirmed PJI	Osteolysis	Loosening	Malalignment/Malposition	Instability	Other
One	26 (45.6%)	17 (54.8%)	2 (8.3%)	0	3 (10.7%)	2 (16.6%)	2 (100%)
Two	13 (22.8%)	8 (25.8%)	4 (16.7%)	1 (5.6%)	10 (35.7%)	3 (25.0%)	0
Three	9 (15.8%)	3 (9.7%)	9 (37.5%)	8 (44.4%)	6 (21.4%)	1 (8.3%)	0
Four	9 (15.8%)	3 (9.7%)	9 (37.5%)	9 (50.0%)	9 (31.1%)	6 (50.0%)	0

Abbreviations: loosening, clinical loosening of the component; malalignment/malposition, joint malalignment or component malposition; other, other isolated causes; PJI, prosthetic joint infection.

**Table 4:** The association between volumetric wear and time in situ, and between volumetric wear and causes of TKA failure.

	Estimate (95% CI)	*p*-Value
Time in situ, months	0.96 (0.21–1.71)	0.013
Confirmed PJI		
Yes	23.96 (−10.9–58.82)	0.174
No	Ref.	
Osteolysis and clinical loosening of a component		
Both causes	41.16 (10.98–71.34)	0.009
Osteolysis alone	52.06 (10.02–94.09)	0.016
No	Ref.	
Joint malalignment or component malposition		
Yes	25.23 (−2.26–52.72)	0.071
No	Ref.	
Instability		
Yes	23.16 (−14.38–60.7)	0.221
No	Ref.	

Abbreviations: CI, confidence interval; Ref., reference.

## Data Availability

The data presented in this study are available upon request from the corresponding author. The data are not publicly available due to technical reasons.
